# Splenic artery embolization in a woman with bleeding gastric varices and splenic vein thrombosis: a case report

**DOI:** 10.1186/1752-1947-4-247

**Published:** 2010-08-04

**Authors:** Bernd Saugel, Jochen Gaa, Veit Phillip, Roland M Schmid, Wolfgang Huber

**Affiliations:** 1 II. Medizinische Klinik und Poliklinik, Klinikum rechts der Isar der Technischen Universität München, München, Deutschland; 2 Institut für Röntgendiagnostik, Klinikum rechts der Isar der Technischen Universität München, München, Deutschland

## Abstract

**Introduction:**

Gastric variceal bleeding due to splenic vein thrombosis is a life-threatening situation and is often difficult to manage by endoscopy. In the worst cases, an emergency splenectomy may be required to stop variceal bleeding.

**Case presentation:**

We report the case of a 60-year-old Caucasian woman with bleeding gastric varices secondary to splenic vein thrombosis treated by splenic artery embolization. Successful embolization was performed by depositing coils into the splenic artery resulting in cessation of variceal bleeding. After embolization there was no recurrence of bleeding.

**Conclusion:**

Splenic artery embolization can be an effective and definite treatment for variceal bleeding secondary to splenic vein thrombosis.

## Introduction

In recent years new endoscopic techniques for the management of active variceal hemorrhage have been introduced [1,2]. Although advances have been made in the treatment of bleeding varices, bleeding from gastric varices can be a life-threatening situation in patients with portal hypertension [3]. Gastric varices are challenging due to the difficulty of endoscopic approach and high recurrence rate [4]. This high recurrence rate is associated with poor prognosis and decreased survival. The cumulative mortality of gastric varices is as high as around 50% at one year [3,5]. The optimal treatment of gastric variceal bleeding remains controversial [6,7]. Different treatment options for gastric variceal bleeding secondary to splenic vein thrombosis have been discussed. Splenectomy was considered the best treatment in the past [8,9]. Recently, splenic artery embolization has been suggested to be an effective method for the treatment of bleeding from gastric varices and portal hypertension [10].

This case report concerns a 60-year-old Caucasian woman with bleeding gastric varices secondary to splenic vein thrombosis treated by partial splenic artery embolization.

## Case presentation

A 60-year-old Caucasian woman was admitted to our hospital because of severe upper gastrointestinal bleeding. An endoscopy was performed, revealing bleeding from gastric varices in the subcardial region. Due to the large variceal size, endoscopic therapy with variceal ligation could not be performed. She was transferred to our intensive care unit (ICU).

She had a history of similar episode of massive gastrointestinal bleeding from gastric varices six years previously. Evaluation at that time with liver function tests, portal venous flow and magnetic resonance angiography did not reveal an identifiable cause.

**Figure 1:**
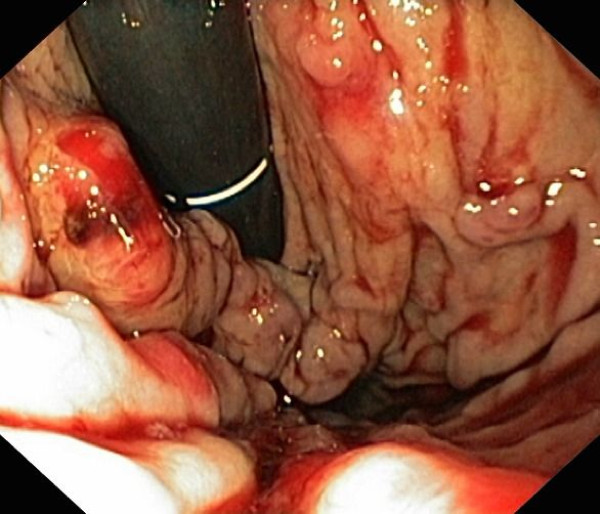
**Bleeding from subcardial gastric varices**. Esophagogastroduodenoscopy revealing bleeding from subcardial gastric varices

On admission to the ICU our patient initially showed no signs of hypovolemic shock. Laboratory results again did not indicate impaired liver function. Esophagogastroduodenoscopy again revealed bleeding from subcardial gastric varices in the absence of evidence of esophageal varices (Figure 1).

**Figure 2:**
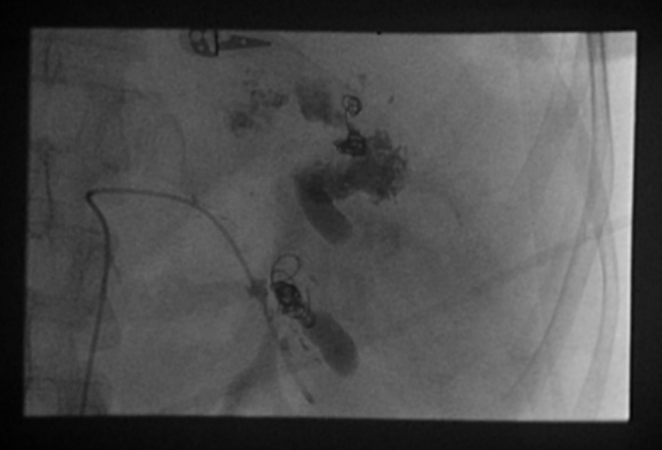
**Emergency splenic artery embolization**. The figure illustrates successful splenic artery embolization via the transcatheter approach after depositing coils into the splenic artery.

Endoscopic treatment with histoacryl glue only resulted in a temporary reduction of the bleeding. To reduce the portal venous pressure the vasopressin analog terlipressin was administered. Despite this therapy, there was another severe episode of upper gastrointestinal bleeding with signs of shock. In all, 10 units of red blood cell concentrate and four units of fresh frozen plasma were transfused. Sufficient endoscopic therapy could not be achieved. An abdominal sonograph showed she had an enlarged spleen (15.9 × 5.4 cm; liver size and structure were normal, with normal flow in the portal vein). A computed tomography (CT) scan showed total occlusion of the splenic vein. Despite the limited data on urgent splenic artery embolization, she was subsequently referred for interventional radiological procedures. A successful splenic artery embolization was performed via the transcatheter approach, depositing coils into the splenic artery resulting in immediate cessation of variceal bleeding (Figure 2).

No recurrence of bleeding was noted post-embolization. After embolization, our patient complained of mild left upper abdominal discomfort, which was effectively relieved by routine analgesics.

At two weeks after admission our patient was fully recovered and was released from our department. There were no further bleeding complications for 18 months. Follow-up endoscopy was performed two months and eight months after the intervention, showing only mild gastric varices without signs of bleeding.

## Conclusions

Our case illustrates that splenic artery embolization can be a quick and effective method of controlling gastric variceal bleeding in patients with portal hypertension associated with splenic vein thrombosis. Splenic artery embolization results in a restriction of blood flow to the spleen and a reduction of transmural pressure and size of gastric varices.

Splenic embolization has the advantage of being a non-operative intervention that can be performed under local anesthesia. Splenic artery embolization has been shown to be an effective alternative to splenectomy with reduced morbidity and mortality [11]. Post-embolization syndrome is the most common side effect of splenic artery embolization [12]. It is a self-limiting, benign phenomenon that usually consists of left abdominal pain, fever, malaise, and gastrointestinal symptoms. Serious complications of this therapeutic method, such as splenic abscess and septicemia, are very rare [13].

In summary, splenic artery embolization can be a quick and effective method of controlling gastric variceal bleeding in patients with portal hypertension associated with splenic vein thrombosis.

## Competing interests

The authors declare that they have no competing interests.

## Authors' contributions

BS wrote the case report. JG was the physician who performed embolization. VP wrote the case report. RMS wrote the case report and gave final approval. WH wrote the case report, was the physician in charge of the ICU and performed the endoscopy. All authors read and approved the final manuscript.

## Consent

Written informed consent was obtained from the patient for publication of this case report and any accompanying images. A copy of the written consent is available for review by the Editor-in-Chief of this journal.
